# Phosphorylation of a chronic pain mutation in the voltage-gated sodium channel Nav1.7 increases voltage sensitivity

**DOI:** 10.1074/jbc.RA120.014288

**Published:** 2020-12-29

**Authors:** Clara M. Kerth, Petra Hautvast, Jannis Körner, Angelika Lampert, Jannis E. Meents

**Affiliations:** 1 Institute of Physiology, Uniklinik RWTH Aachen University, Aachen, Germany; 2 Department of Anesthesiology, Uniklinik RWTH Aachen University, Aachen, Germany

**Keywords:** sodium channel, pain, phosphorylation, protein kinase C (PKC), protein kinase A (PKA), patch clamp, erythromelalgia, Nav1.7, I848T, CC, calphostin C, CHO-K1, Chinese hamster ovary cell line, CI, confidence interval, Diff., difference, E_rev_, reversal potential, HEK, human embryonic kidney cell line, IEM, inherited erythromelalgia, *k*, slope factor, Nav, voltage-gated sodium channel, PKA, protein kinase A, PKC, protein kinase C, PMA, phorbol 12-myristate 13-acetate, S, staurosporine, V_1/2_, membrane potential at half-maximal activation/inactivation, V_m_, membrane potential

## Abstract

Mutations in voltage-gated sodium channels (Navs) can cause alterations in pain sensation, such as chronic pain diseases like inherited erythromelalgia. The mutation causing inherited erythromelalgia, Nav1.7 p.I848T, is known to induce a hyperpolarized shift in the voltage dependence of activation in Nav1.7. So far, however, the mechanism to explain this increase in voltage sensitivity remains unknown. In the present study, we show that phosphorylation of the newly introduced Thr residue explains the functional change. We expressed wildtype human Nav1.7, the I848T mutant, or other mutations in HEK293T cells and performed whole-cell patch-clamp electrophysiology. As the insertion of a Thr residue potentially creates a novel phosphorylation site for Ser/Thr kinases and because Nav1.7 had been shown in *Xenopus* oocytes to be affected by protein kinases C and A, we used different nonselective and selective kinase inhibitors and activators to test the effect of phosphorylation on Nav1.7 in a human system. We identify protein kinase C, but not protein kinase A, to be responsible for the phosphorylation of T848 and thereby for the shift in voltage sensitivity. Introducing a negatively charged amino acid instead of the putative phosphorylation site mimics the effect on voltage gating to a lesser extent. 3D modeling using the published cryo-EM structure of human Nav1.7 showed that introduction of this negatively charged site seems to alter the interaction of this residue with the surrounding amino acids and thus to influence channel function. These results could provide new opportunities for the development of novel treatment options for patients with chronic pain.

Voltage-gated sodium channels (Navs) are important for the proper functioning of our body, especially in the nervous system and muscle tissue including cardiac muscle cells. Human Navs have four pore-forming domains (DI–IV), each of them equipped with six transmembrane segments (S1–S6). S1–S4 of each domain contain the voltage sensor, whereas S5/S6 form the channel pore (1, 2). Nine human Nav isoforms (Nav1.1–1.9) have been described (3), expressed throughout different tissues (1, 4, 5). Next to the pore-forming α-subunit of Nav channels, there are four β-subunits (SCNB1b–4b; β1–4), which are believed to modify the gating and expression of Navs (1, 5). Among the nine Nav isoforms, Nav1.7 to 1.9 are relevant for the perception of pain as they are mainly located in peripheral sensory neurons of the dorsal root ganglion, sympathetic ganglion, and in olfactory sensory neurons (6, 7, 8, 9).

In a previous study, we have shown that human Nav1.7 is especially important for defining the threshold of the action potential in nociceptors and plays an important role in the action potential upstroke (6). Considering this role of Nav1.7, one might deduct that mutations in Nav1.7 can lead to changes in neuronal excitability and thereby to altered pain perception. Accordingly, several mutations in Nav1.7 are known to cause a variety of chronic pain syndromes, such as inherited erythromelalgia (IEM) or paroxysmal extreme pain disorder, and seem to play a role in small fiber neuropathy (7, 10).

Patients with IEM have sudden pain attacks and synchronous erythema of the distal extremities, often triggered by exercise or exposure to higher temperatures (11, 12). Unfortunately, neither common pain treatments nor nonselective sodium channel blockers, such as lidocaine, provide satisfactory pain relief (7, 13, 14). One selective sodium channel blocker (PF-0508977) was described to mildly relieve pain in patients with IEM, but the effects were very short lasting (15).

Different gain-of-function mutations known to cause IEM are located in the SCN9A gene, which encodes for Nav1.7 (16, 17, 18, 19, 20, 21, 22). One of the earliest known of such mutations is the gain-of-function mutation Nav1.7 p.I848T, which is located in the intracellular S4–S5 linker region of domain II (Fig. 1*A*) (16, 17) and which leads to an early onset of IEM (11, 17, 19). Throughout all domains, the S4–S5 linker regions are known to contain a variety of disease- (and IEM-) causing mutations in Navs (14, 16, 23, 24, 25, 26, 27, 28). The functional importance of the S4–S5 linker regions for Nav channel gating has recently been shown using cryo-EM of the ancestral bacterial Nav channel (29). Of interest, the authors identified three amino acid residues that are key for the opening and closing of the channel gate: I119, L123, V126 (29). These data might suggest that any changes to these crucial amino acids could affect Nav gating and nociceptor excitability. The human Nav1.7 I848 residue corresponds to the crucial amino acid L123 of the bacterial Nav channel. This could explain why this mutation causes changes in the gating of Nav1.7. In fact, we and others have shown that the Nav1.7/I848T mutation induces significant changes in action potential firing behavior of human induced pluripotent stem cell–derived nociceptors, such as a decreased firing threshold, a decreased time to peak, and an enhanced upstroke of the action potential (6, 15).

**Figure 1:**
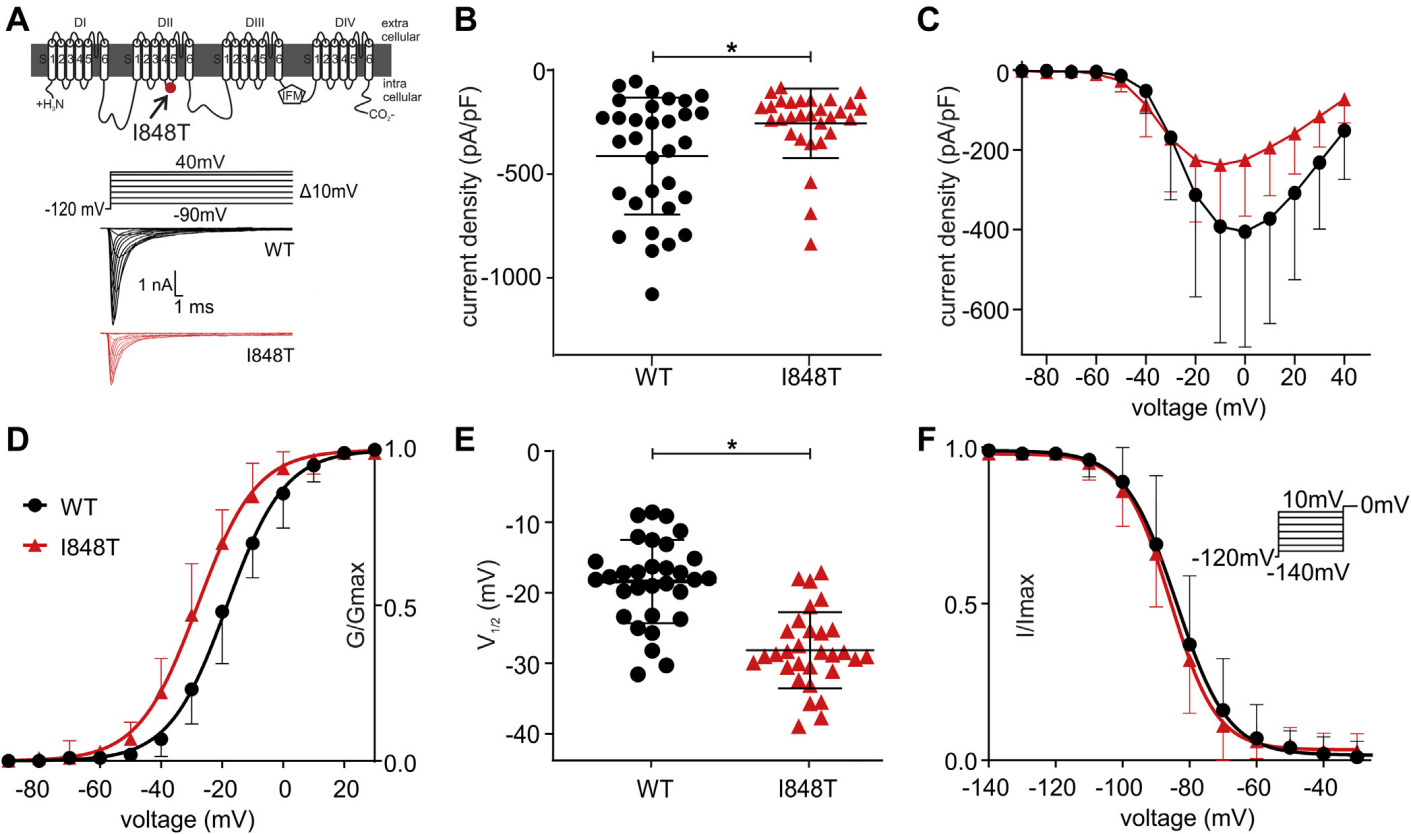
**The Nav1.7/I848T mutation changes the voltage dependence for activation but not for inactivation**. *A*, schematic representation of Nav1.7 with the location of the I848T mutation, which leads to inherited erythromelalgia. The voltage protocol of activation (top) with two representative traces for the Nav1.7/WT (middle, *black*) and I848T mutant (bottom, *red*). *B*, the I848T mutant produces significantly less current than Nav1.7/WT (*p* = 0.04 Mann–Whitney test, median diff. −122.7 pA/pF, 95.11% confidence interval 3.79–248.8 pA/pF). *C*, current–voltage relationship shown for WT and I848T mutant. *D*, the I848T mutant leads to a hyperpolarized shift of −9.77 ± 1.53 mV in the voltage dependence of activation of Nav1.7. *E*, values of half-maximal voltage dependence of activation (V_1/2_) for WT and I848T mutant (*p* < 0.0001, t = 6.61, df = 59, unpaired *t* test, mean diff. −9.73 ± 1.45 mV, 95% confidence interval −12.67 to −6.86 mV). *F*, there is no difference in the voltage dependence of steady-state fast inactivation for the I848T mutant. The applied voltage protocol is shown in the *inset*. See Table 1 for all values. All data are presented as mean ± SD.

Phosphorylation can modify and regulate the function or expression of Nav channels (2, 30, 31, 32, 33, 34). It was shown in *Xenopus laevis* oocytes that phosphorylation by protein kinase C (PKC) and protein kinase A (PKA) can increase or decrease the peak currents of Nav1.7 and Nav1.8 and more importantly induce shifts in voltage dependence of activation (30). In this light, it seems possible that phosphorylation might be responsible for the changes of the Nav1.7/I848T mutation, as this mutant provides a potential novel phosphorylation site.

In this study, we show in whole-cell voltage-clamp experiments that phosphorylation of the Nav1.7/I848T mutant is responsible for the hyperpolarized shift in the voltage dependence of activation. Moreover, by introducing a negative charge to mimic the phosphorylated I848T residue, we confirm a possible phosphorylation. To try to close the gap between the newly discovered phosphorylation of the I848T mutant and its effect on channel gating, we performed mutagenesis to investigate potential molecular interactions of the phosphorylated side group and the channel pore. We show that this negatively charged side chain could interfere with noncovalent π-interactions of the surrounding phenylalanines and could thereby lead to changes in channel gating. In the following sections, the mutant Nav1.7 channel will be termed I848T, whereas the individual mutated amino acid residue will be labeled T848.

## Results

### The I848T mutation causes a hyperpolarized shift in voltage dependence of activation

The gain-of-function mutation Nav1.7/I848T (Fig. 1*A*) is known to cause IEM. Earlier studies have shown that the I848T mutation causes a hyperpolarizing shift in the voltage dependence of activation of Nav1.7 (16), which seems to lead to increased pain in the patient (6, 15, 17). Here, we first attempted to confirm these earlier results in a heterologous expression system, using human embryonic kidney cell line HEK293T cells transiently transfected with either wildtype Nav1.7 or its I848T mutant form.

First, we found that the I848T mutant produces significantly smaller currents than the wildtype channel (Fig. 1, *B*–C, Table 1), which has not been reported so far. An earlier study of Cummins *et al*. (17) cotransfecting Nav1.7 with the β1- and β2-subunits did not show the decrease in current density. However, when we cotransfected both β-subunits with the I848T mutant or the wildtype channel, we still observed a significant decrease in current density for the I848T mutant (Fig. S1*C* and Table S1). Furthermore, we identified a hyperpolarized shift in the voltage dependence of activation of −9.77 ± 1.53 mV (Fig. 1, *D–E*, Table 1), which is comparable with earlier results reported in heterologous systems (7, 16, 17) as well as to previous results in iPS-derived nociceptors of patients with IEM (6). As expected, we did not find any difference in the voltage dependence of steady-state fast inactivation between the wildtype and I848T (Fig. 1*F*).

**Table 1:** Voltage-clamp parameters of the different Nav1.7 mutants

Genotype	Activation				Fast inactivation			Current density
	n	V_1/2_ (mV)	Slope (V/s)	Gmax (pS)	n	V_1/2_ (mV)	Slope (V/s)	(pA/pF)
WT	31	−18.3 ± 5.91	8.91 ± 1.24	76.62 ± 53.13	30	−84.21 ± 6.82	6.67 ± 1.42	−416.5 ± 291.5
I848T	30	−28.06 ± 5.42	8.99 ± 1.48	41.01 ± 29.51	32	−86.32 ± 5.11	6.48 ± 1.36	−252.8 ± 167.6
I848E	20	−24.1 ± 6.91	11.09 ± 1.77	7.79 ± 4.59	15	−84.05 ± 6.6	6.99 ± 1.93	−53.29 ± 47.09
F1432L WT	18	−20.02 ± 4.03	9.14 ± 0.83	110.0 ± 91.61	20	−83.82 ± 4.07	6.70 ± 1.55	−340.4 ± 229
F1432L IT	14	−40.76 ± 7.91	9.9 ± 2.03	15.89 ± 13.42	9	−86.02 ± 4.69	5.59 ± 1.11	−91.68 ± 53.48
F832L WT	14	−18.24 ± 5.42	9.24 ± 1.14	124.7 ± 86.09	11	−84.06 ± 7.23	6.58 ± 1.12	−445.6 ± 315.4
F832L IT	14	−25.7 ± 5.13	8.94 ± 1.26	72.34 ± 50.26	11	−82.89 ± 4.93	6.13 ± 0.74	−406.9 ± 246.9
F1320L WT	9	−15.94 ± 5.7	8.64 ± 1.27	124.6 ± 93.52	9	−85.97 ± 4.03	5.9 ± 1.18	−550.4 ± 427
F1320L IT	14	−25.70 ± 6.2	9.82 ± 1.26	81.79 ± 46.08	19	−84.37 ± 4.7	6.38 ± 1.12	−278.2 ± 80.83
F1436L WT	15	−19.20 ± 3.62	8.94 ± 1.37	69.16 ± 49.75	9	−73.96 ± 3.51	5.59 ± 1.39	−194.3 ± 107.6
F1436L IT	17	−29.91 ± 2.5	9.21 ± 1.23	34.21 ± 27.26	13	−74.40 ± 4.14	5.93 ± 0.79	−111.0 ± 64.26

All data are presented as mean ± SD.

With these experiments, we were able to confirm the gain-of-function nature of the I848T mutation (6, 16, 17). However, it is not clear which mechanism underlies the mutation-induced hyperpolarized shift of activation of Nav1.7.

### Phosphorylation explains the hyperpolarized shift in activation

**Figure 2:**
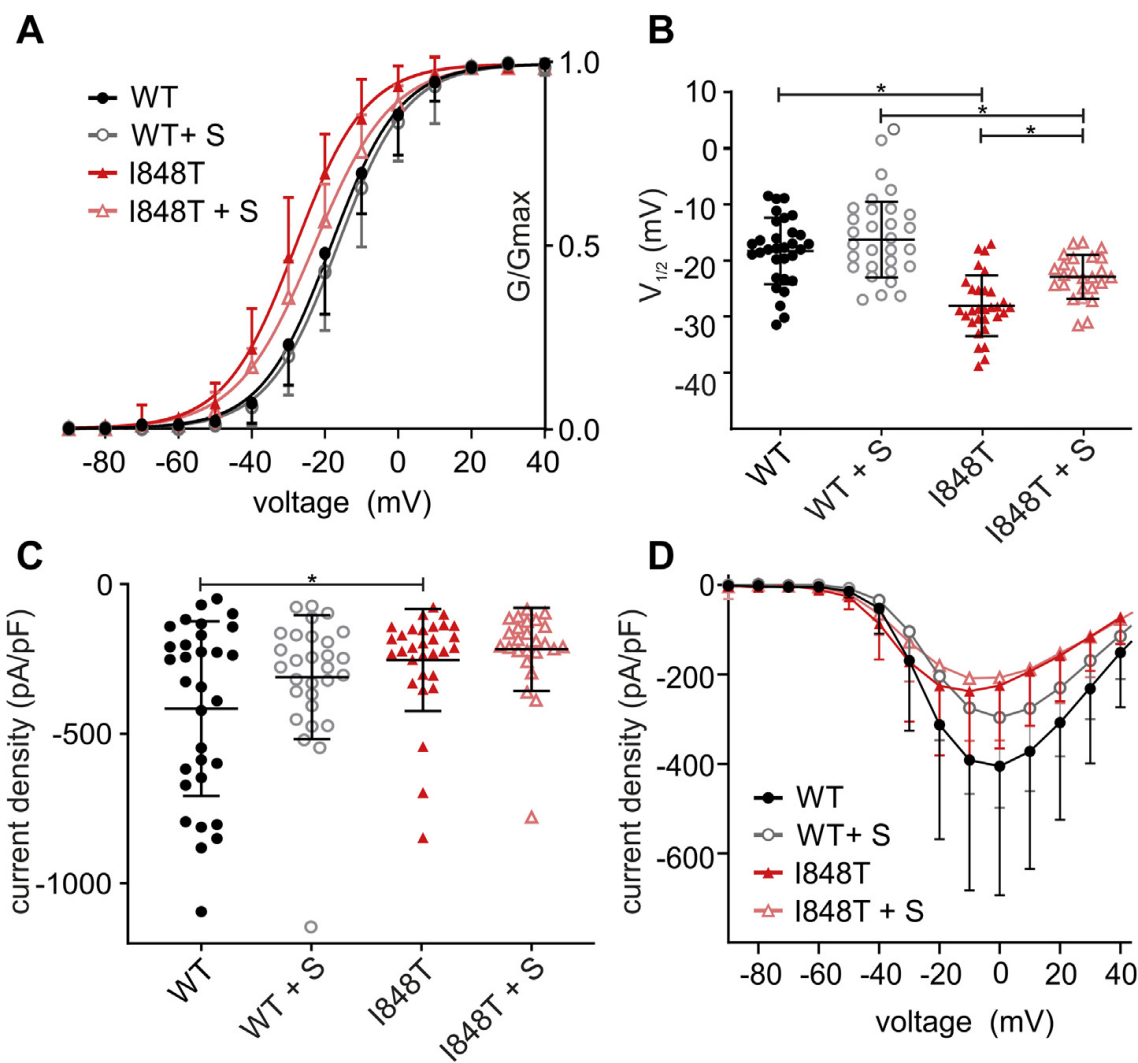
**The nonspecific kinase inhibitor staurosporine reduces the hyperpolarized shift of the I848T mutation**. *A*, voltage dependence of activation using 500 nM staurosporine, incubated for 20 min. Staurosporine reduces the hyperpolarized shift of the I848T mutation from −9.77 ± 1.45 to −6.67 ± 1.54 mV. *B*, V_1/2_ of channel activation (ANOVA F = 25.67; WT *versus* I848T *p* <0.0001, mean diff. 9.77 ± 1.45 mV, 95% confidence interval [CI] 5.88–13.65 mV; WT+S *versus* I848T + S *p* = 0.0002, mean diff. 6.67 ± 1.54 mV, 95% CI 2.53–10.80 mV; I848T *versus* I848T + S *p* = 0.0064, mean diff. −5.14 ± 1.529 mV, 95% CI −9.247 to −1.032 mV). *C*, staurosporine does not significantly affect current density (ANOVA F = 4.8; WT *versus* I848T *p* = 0.0213, mean diff. −162.9 ± 54.68 pA/pF, 95% CI −309.8 to −15.99 pA/pF). *D*, current–voltage relationship with or without staurosporine. Data for WT and I848T are the same as for Figure 1 and only presented for comparison. All are one-way ANOVA with Bonferroni multiple comparisons test. See Tables 1 and 2 for all values. All data are presented as mean ± SD. WT + S = WT + staurosporine, I848T + S = I848T mutant + staurosporine.

The I848T substitution creates a novel potential phosphorylation site for threonine kinases since it replaces an isoleucine with threonine, the typical substrate for this class of kinases. Owing to its intracellular location, a phosphorylation at this position seems reasonable (Fig. 1*A*). This implies that the hyperpolarized activation curve of the I848T mutant could be induced by phosphorylation. We used staurosporine, a nonspecific protein kinase inhibitor (23), to reduce phosphorylation and thereby potentially counteract the disease-causing effect of the I848T mutant. Indeed, staurosporine led to a significant reduction of the hyperpolarized shift in voltage dependence of activation for the I848T mutant (Fig. 2, *A*–*B* and Table 2). The activation curve of the staurosporine-treated I848T mutant displayed a shallower slope than the other curves (Fig. 2*A* and Table 2), which could suggest a mixed population of phosphorylated and unphosphorylated channels. However, increasing the concentration of staurosporine had no further effect on voltage dependence or on the slope factor (Table 2). It is therefore possible that nonspecific kinase inhibitors are not effective enough in abolishing phosphorylation of the I848T mutant. Considering that staurosporine had no effect on the voltage dependence of activation for the wildtype channel (Fig. 2 and Table 2), it is interesting to note that protein kinase–mediated phosphorylation does not seem to play an important role for the gating of the unmutated Nav1.7.

**Table 2:** Voltage-clamp parameters for Nav1.7 wildtype and I848T mutant treated with different kinase inhibitors and activators

Genotype	Activation				Fast inactivation			Current density
	n	V_1/2_ (mV)	Slope (V/s)	Gmax (pS)	n	V_1/2_ (mV)	Slope (V/s)	(pA/pF)
WT	31	−18.3 ± 5.91	8.91 ± 1.24	76.62 ± 53.13	30	−84.21 ± 6.82	6.67 ± 1.42	−416.5 ± 291.5
I848T (IT)	30	−28.06 ± 5.42	8.99 ± 1.48	41.01 ± 29.51	32	−86.32 ± 5.11	6.48 ± 1.36	−252.8 ± 167.6
WT+ 500 nM staurosporine	29	−16.26 ± 6.76	8.8 ± 1.12	62.35 ± 51.54	24	−88.74 ± 5.31	6.79 ± 1.24	−310.6 ± 207.3
IT + 500 nM staurosporine	25	−22.92 ± 3.89	10.4 ± 1.64	37.41 ± 28.18	22	−86.4 ± 5.96	6.57 ± 0.99	−217.7 ± 139.1
IT + 1 μM staurosporine	19	−24.74 ± 4.77	10.25 ± 0.92					
WT + 200 nM calphostin C	24	−16.57 ± 6.35	8.15 ± 1.25	102.3 ± 93.13	25	−83.68 ± 6.08	7.88 ± 1.39	−399.6 ± 420.4
IT + 200 nM calphostin C	25	−20.90 ± 4.97	9.55 ± 1.41	57.77 ± 54.35	21	−81.05 ± 5.72	8.59 ± 1.9	−216.1 ± 215.5
WT + 1 μM PMA	24	−18.54 ± 3.61	8.44 ± 1.34	86.69 ± 63.81	28	−85.02 ± 2.3	6.58 ± 1.13	−425.6 ± 288.9
IT + 1 μM PMA	31	−25.84 ± 4	9.77 ± 1.33	57.84 ± 39.13	27	−83.47 ± 5.23	6.29 ± 1.06	−265.9 ± 161.2
WT + 10 μM H-89	25	−16.87 ± 4.96	9.21 ± 1.32	63.74 ± 39.8	21	−85.91 ± 5.04	6.72 ± 0.83	−327.4 ± 169.6
IT + 10 μM H-89	25	−25.39 ± 4.49	10.02 ± 1.5	35.04 ± 7.56	25	−86.55 ± 4.43	6.54 ± 0.8	−178.6 ± 111.2

All data are presented as mean ± SD. Staurosporine is a nonspecific kinase inhibitor. Calphostin C is a selective protein kinase C inhibitor. PMA (phorbol 12-myristate 13-acetate) is a protein kinase C activator. H-89 is a selective protein kinase A inhibitor.

These results using staurosporine suggest that phosphorylation of I848T might explain at least some of the hyperpolarized shift in voltage dependence of activation, which seems to cause the gain of function in Nav1.7.

### A negative side group at position T848 supports a possible phosphorylation

The results with staurosporine suggest that phosphorylation of the I848T mutant could be responsible for the hyperpolarized shift in the voltage dependence of activation. To support these results, we replaced threonine by the negatively charged amino acids glutamate (I848E) and aspartate (I848D), which mimic the negative charge of the putative phosphothreonine (34, 35, 36). Therefore, both mutations (I848E and I848D) should have a similar effect as the phosphorylated I848T mutant and show a hyperpolarized shift in the voltage dependence of activation.

**Figure 3:**
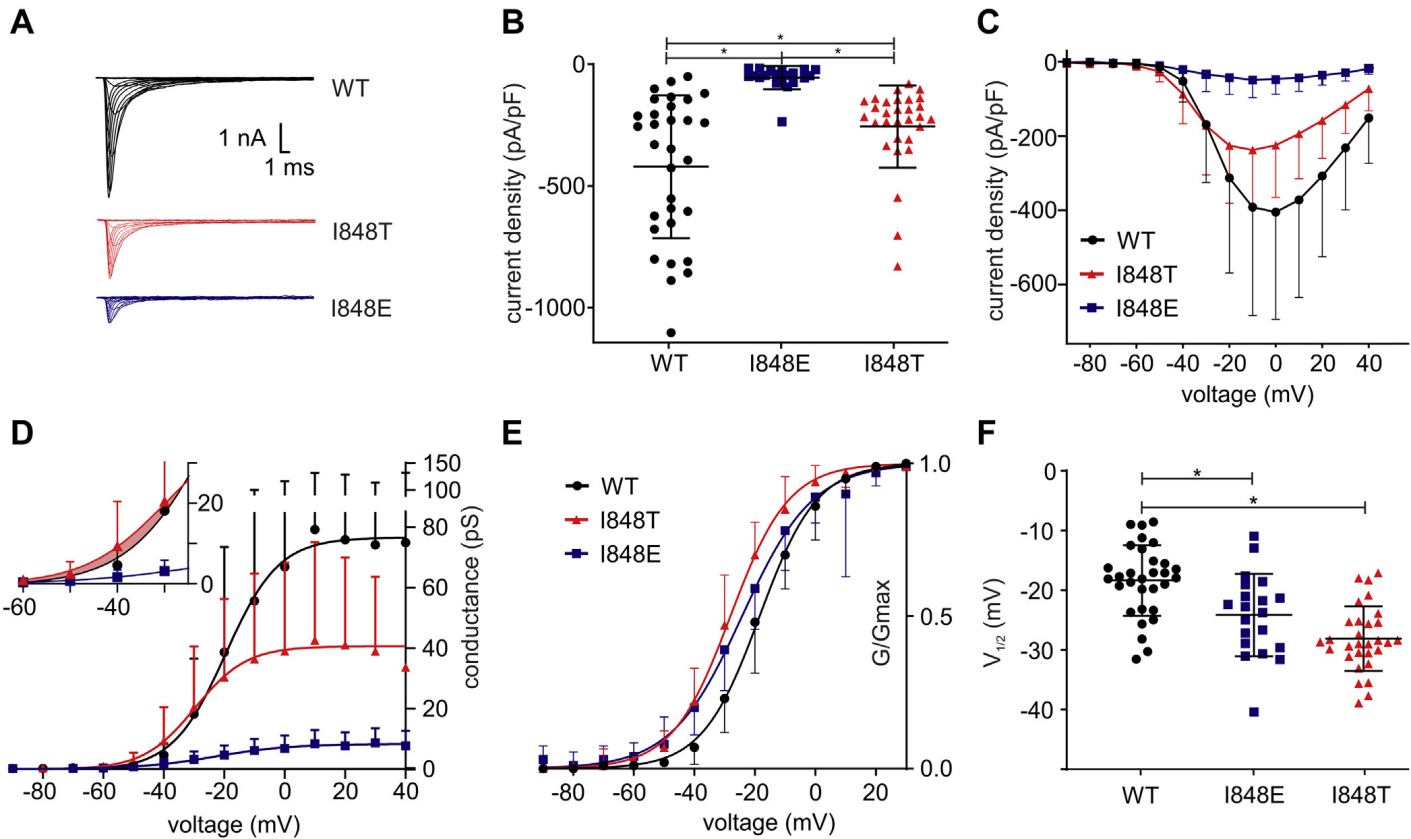
**The mutant I848E presents a similar shift in voltage dependence of activation in Nav1.7**. *A* and *B*, the negatively charged mutant I848E (*blue*) exhibited much smaller currents (*A*) and a significantly reduced current density (*B*) when compared with wildtype Nav1.7 or the putatively phosphorylated I848T mutation (ANOVA F= 18.53; WT *versus* I848E *p* < 0.0001, mean diff. −363.2 ± 60.18 pA/pF, 95% confidence interval [CI] −510.4 to 215.9 pA/pF; WT *versus* I848T *p* = 0.0099, mean diff. −162.9 ± 53.74 pA/pF, 95% CI −294.4 to 31.39 pA/pF; I848E *versus* I848T *p* = 0.0040, mean diff. −200.3 ± 60.57 pA/pF, 95% CI 52.09–348.5 pA/pF). *C*, current-voltage relationship for WT, I848T and I848E. *D*, raw conductance of WT, I848T and I848E. The *inset* shows that at negative voltages relevant for neuronal activity, conductance is slightly increased for the I848T mutant (*shaded area*). *E*, I848T and I848E mutant channels display a hyperpolarized voltage dependence of activation. *F*, the V_1/2_ of activation is significantly reduced in both mutant channels (ANOVA F= 19.83; WT *versus* I848E *p* = 0.0036, mean diff. 5.8 ± 1.72 mV, 95% CI 1.59–10.01 mV; WT *versus* I848T *p* <0.0001, mean diff. 9.76 ± 1.54 mV, 95% CI 6.0–13.52 mV). Data for WT and I848T are the same as for Figure 1 and only presented for comparison. All are one-way ANOVA with Bonferroni multiple comparisons test. See Tables 1 and 2 for all values. All data are presented as mean ± SD.

We were not able to record any current from cells transfected with the I848D mutation, suggesting that the mutation renders the channel nonfunctional. The I848E mutant, on the other hand, was functional, although we noticed a strong decrease in the current density compared with the I848T mutant and wildtype (Fig. 3, *A*–*C* and Table 1). This decrease in current density could not be reversed with cotransfection of β1- and β2-subunits (Fig. S1*C* and Table S1). The reduced current density of cells expressing I848E and I848T channels could be due to the negative charge at this residue, caused either by the I848E mutant or by the phosphorylation of the I848T.

It is difficult to reconcile a loss-of-function effect, such as a loss in current density, with a gain-of-function shift in voltage gating that we have seen for the I848T mutation. We therefore plotted the raw conductance of wildtype, I848T, and I848E channels (Fig. 3*D*) in order to visualize and compare both effects. For the I848E mutant, the conductance across all voltages was very low in line with the dramatically reduced current density. However, in case of the I848T mutant, the conductance at negative voltages around the activation threshold of Nav1.7 was slightly and nonsignificantly increased as compared with wildtype (shaded area in Fig. 3*D*). This suggests that, at voltages around the action potential threshold (6), the shift in voltage activation dominates the reduced current density.

In order to better visualize a potential shift in voltage activation for the I848E mutation, we again normalized conductance values (Fig. 3*E*). Cells expressing the I848E mutant show a hyperpolarized shift in the voltage dependence of activation of −5.8 ± 1.72 mV as compared with wildtype (Fig. 3, *E*–*F* and Table 1). Similar results were also obtained when cotransfected with β1- and β2-subunits (Fig. S1, *A–B* and Table S1). Therefore, the I848E mutant shows a similar behavior as the I848T mutant, which could indicate that the latter is phosphorylated and therefore also negatively charged.

In summary, these results with a negative charged residue at T848 support our previous findings of a putative phosphorylation of the I848T residue, which induces a hyperpolarizing shift in activation of the mutated Nav1.7.

### Phosphorylation of I848T is mediated by PKC but not PKA

**Figure 4:**
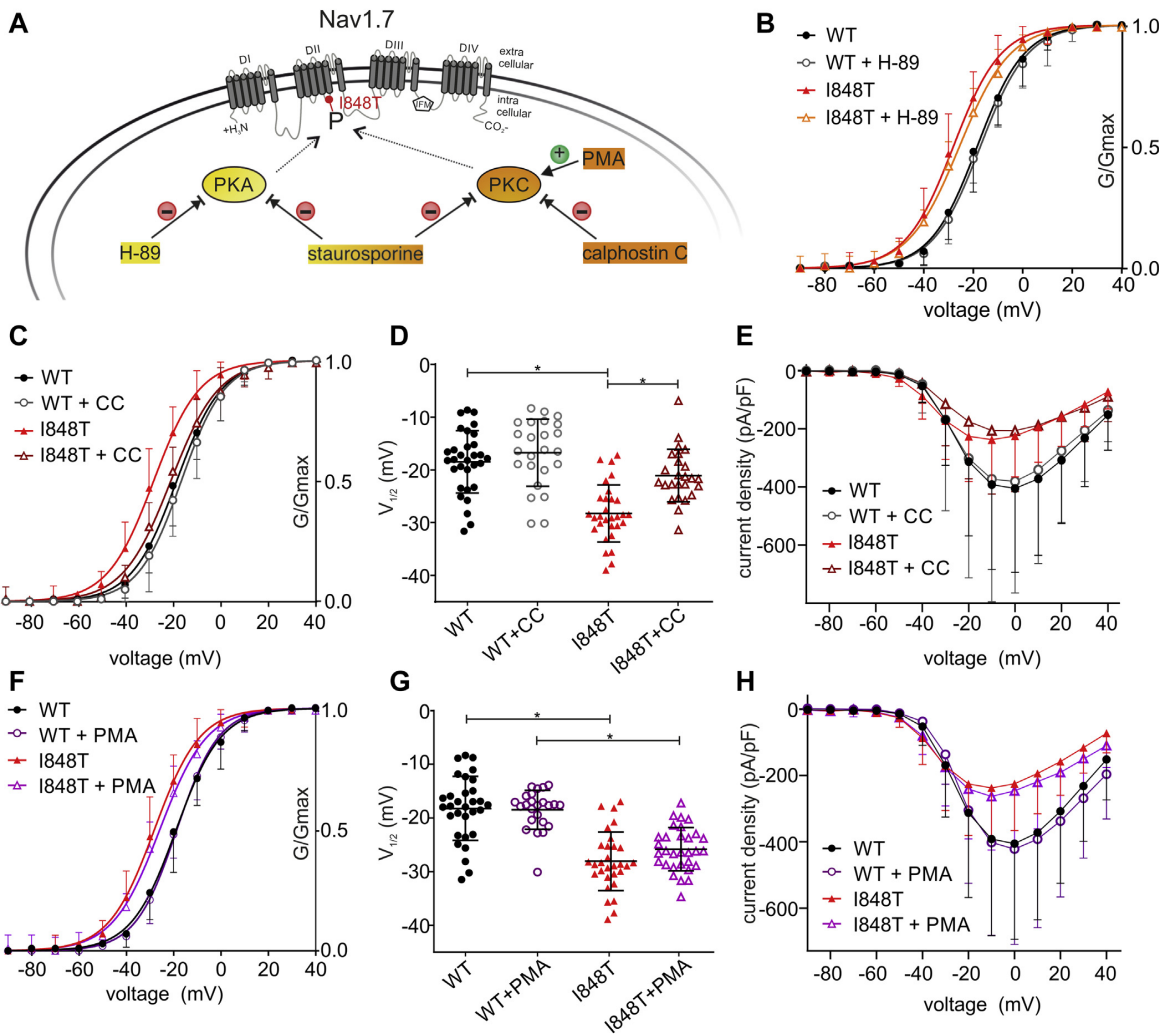
**PKC but not PKA is responsible for the phosphorylation of I848T**. *A*, schematic overview of the applied activators and inhibitors of phosphorylation: *green circle*, activator; *red circle*, inhibitor. *B*, the PKA inhibitor H-89 (10 μM) does not affect the voltage dependence of activation. *C*, the selective PKC inhibitor calphostin C (200 nM) almost fully abolishes the hyperpolarized shift of the I848T mutant. See also Figure S7. *D*, values of half-maximal voltage dependence of activation (V_1/2_) for WT and I848T mutant treated with calphostin C (ANOVA F = 22.63; WT *versus* I848T *p* <0.0001, mean diff. −9.77 ± 1.46 mV, 95% confidence interval [CI] 5.86–13.68 mV; I848T *versus* I848T+calphostin C *p* <0.0001, mean diff. −7.16 ± 1.54 mV, 95% CI −11.29 to 3.02 mV). See also Figure S7. *E*, current–voltage relationship shown for I848T and WT with and without calphostin C. See also Figure S7. *F*, PMA (1 μM), a PKC activator, does not further increase the hyperpolarized shift in voltage dependence of activation. *G*, values of V_1/2_ of activation for I848T and WT incubated with and without PMA (ANOVA F = 30.4, WT *versus* I848T *p* <0.0001, mean diff. 9.77 ± 1.25 mV, 95% CI 6.4–13.13. mV; WT+PMA *versus* I848T+PMA *p* <0.0001, mean diff. 7.3 ± 1.33 mV, 95% CI 3.73–10.87 mV). *H*, PMA does not affect current density for WT and I848T. Data for WT and I848T are the same as for Figure 1 and only presented for comparison. All are one-way ANOVA with Bonferroni multiple comparisons test. See Tables 1 and 2 for all values. All data are presented as mean ± SD. CC, calphostin C; I848T, putatively phosphorylated I848T residue in Nav1.7; P, phosphate; PKA, protein kinase A; PKC, protein kinase C; PMA, phorbol 12-myristate 13-acetate.

We next sought to determine which kinases are most likely to be involved in the phosphorylation of I848T. For the phosphorylation of voltage-gated sodium channels, two groups of kinases seem to be particularly relevant: PKC and PKA (30, 31, 32, 33). Therefore, we focused on those two kinases in our experiments. We used different inhibitors and activators for either PKA or PKC (Fig. 4*A*).

First, we checked a possible phosphorylation by PKA using the PKA-specific inhibitor H-89. The prominent shift in voltage dependence of activation for the I848T mutant compared with wildtype was still present when cells were incubated with H-89 (Fig. 4*B* and Table 2). There was no change in voltage dependence of fast inactivation (Fig. S2*F*) and we also did not observe any significant difference in current density (Table 2). Therefore, we can assume that PKA does not mediate the phosphorylation of the I848T mutant Nav1.7 channel and also leaves the wildtype unchanged.

Next, we used calphostin C as a more specific inhibitor of PKC. Calphostin C was able to almost fully abolish the hyperpolarized shift of the I848T mutant in voltage dependence of activation (Fig. 4, *C*–*D* and Table 2) and was therefore more effective than staurosporine in abolishing the disease-causing hypersensitivity of Nav1.7.

It is surprising that the inhibition of PKC seemed to shift fast inactivation for the I848T mutant to a slightly more depolarized potential compared with wildtype (Fig. S2, *C–D* and Table 2). Calphostin C did not reduce the current density of mutant nor wildtype Nav1.7 (Fig. 4*E*, Fig. S3*A* and Table 2).

The above experiment suggests that PKC is responsible for the hyperpolarizing shift in voltage dependence of activation of the I848T mutant. If this is indeed the case, then increasing the activity of PKC, rather than inhibiting it, might increase the voltage sensitivity of the channel even further. We therefore added the PKC activator phorbol 12-myristate 13-acetate (PMA). However, we were not able to show an increase of the hyperpolarizing shift in activation (Fig. 4, *F*–*G* and Table 2). This could be because most of the mutant channels were already phosphorylated. There was also no difference in slope, current density, or fast inactivation (Fig. 4*H*, Fig. S2*E*, Fig. S3*B* and Table 2).

Taken together, these results suggest that PKC but not PKA is responsible for the phosphorylation of Nav1.7/I848T, which leads to the hyperpolarized shift in voltage dependence of activation.

### Possible interactions of phenylalanines with the phosphorylated T848

In order to further investigate the role of a charged side group in the hyperpolarized voltage dependence of activation, we replaced the negative charge at position 848 with a positive charge by inserting a lysine (I848K) or arginine (I848R) residue, hypothesizing that the hyperpolarized shift in voltage dependence would disappear. These two mutations rendered the channel completely nonfunctional and did not conduct any current. This result might suggest that charged residues interact with neighboring amino acids in either the voltage sensor or the channel pore and therefore affect channel gating.

**Figure 5:**
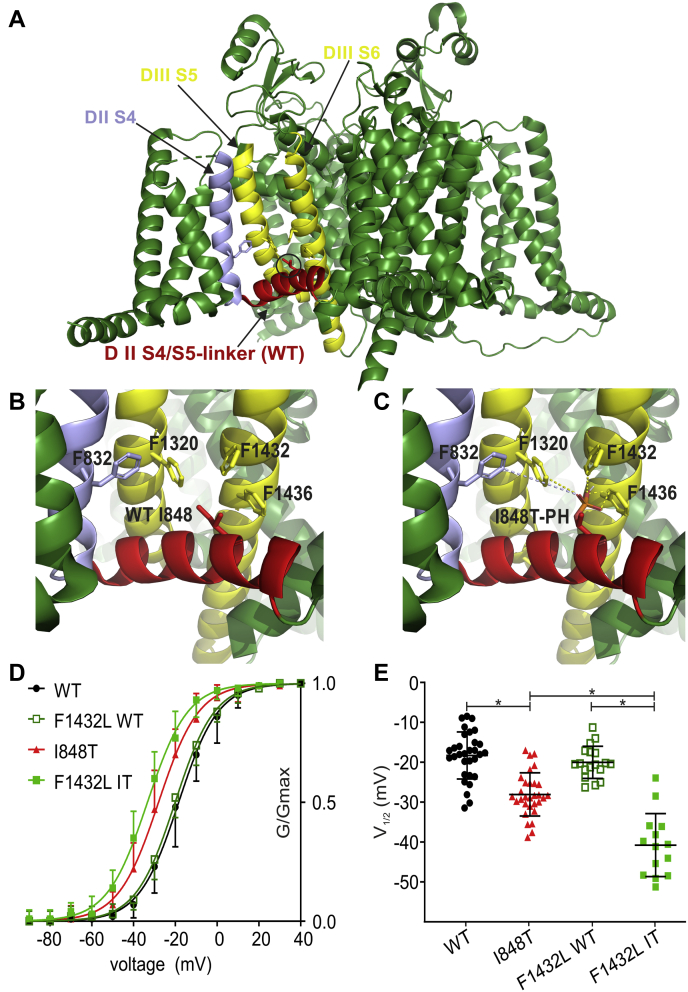
**The I848T residue may interact with the surrounding phenylalanines**. *A*, overview of the 3D structure of the hNav1.7/WT channel. Relevant segments are color coded: S4–S5 linker (*red*); DIIS4 (*blue*); DIIIS5 and DIIIS6 (*yellow*). *B*, magnified view of the area around the I848 residue. Four phenylalanines can be observed in close proximity: F832, F1320, F1432, and F1436. *C*, same view as in (*B*). The I848 residue has been mutated to threonine and a phosphate group has been added to the threonine side chain (I848T-PH). For distances of the surrounding phenylalanines to the phosphate group, see Figure S4*D*. *D*, voltage dependence of activation for the F1432L mutant inserted in a WT (F1432L WT) or I848T (F1432L IT) background. The F1432L IT double mutant induces a greater hyperpolarization of the voltage dependence of activation. *E*, mid-point of activation for single and double mutants (ANOVA F = 45.66, WT *versus* I848T *p* < 0.0001, mean diff. 9.77 ± 1.57 mV, 95% confidence interval [CI] 5.54–13.99 mV; I848T *versus* F1432L IT *p* < 0.0001, mean diff. 9.56 ± 1.8 mV, 95% CI 4.72–14.40 mV; F1432L WT *versus* F1432L IT *p* < 0.0001, mean diff. 17.6 ± 2.01 mV, 95% CI 12.17–23.03 mV). Color coding is the same as for (*D*). Data for WT and I848T are the same as for Figure 1 and only presented for comparison. All are one-way ANOVA with Bonferroni multiple comparisons test. See Tables 1 and 2 for all values and Figure S6 for current–voltage relationship and current density. All data are presented as mean ± SD.

**Figure 6:**
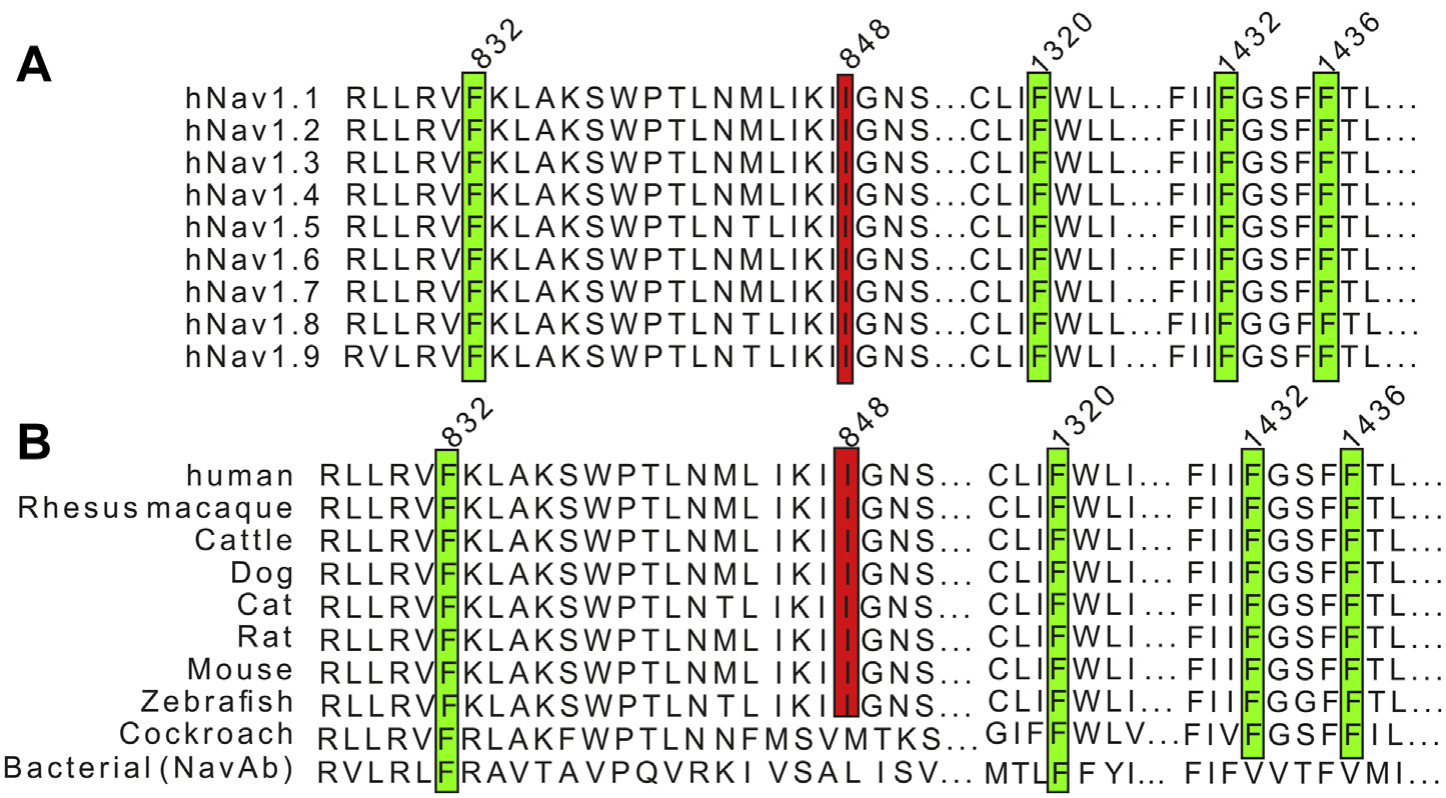
**The I848 and four phenylalanine residues are highly conserved**. Alignment of different Nav isoforms, outlining the importance of I848 and of the four phenylalanines. *A*, alignment of all human Nav isoforms 1.1 to 1.9. *B*, alignment of Nav1.7 throughout different species together with related nonvertebrate channels from cockroach (NavPas) and bacteria (NavAb). See Experimental procedures for details.

To investigate this possibility, we performed 3D modeling using the recently published cryo-EM structure of human Nav1.7 (37). We noticed four phenylalanines in close proximity to the predicted phosphate group at T848 (Fig. 5, *A*–*C* and Fig. S4, *C–D*). Those four phenylalanines are F832 (DIIS4), F1320 (DIIIS5), F1432, and F1436 (both DIIIS6). All four phenylalanine residues as well as the I848 residue are highly conserved across all human Nav channel isoforms and across mammalian and nonmammalian species (Fig. 6), suggesting an important role in channel gating. The distance between each phenylalanine and the predicted phosphate group was between 2.2 to 7.5 Å (Fig. S4*D*), which is close enough for a possible interaction (38). The benzene rings of phenylalanines and similar amino acids are known to undergo several π-interactions (39). Accordingly, we hypothesized that the predicted phosphate group at T848 undergoes a noncovalent π-interaction with one of the above phenylalanines.

Using site-directed mutagenesis, we replaced all four phenylalanines individually with a leucine (F832L, F1320L, F1432L, F1436L) to remove the benzene ring and thereby prohibit the possible π-interaction between the phosphorylated T848 and the phenylalanine. As a control we inserted the same mutations into the Nav1.7 wildtype channel, where no phosphorylation can occur at position I848. Of interest, none of the FL mutations had any effect on the wildtype channel, suggesting that each individual benzene ring might not be essential for channel function (Fig. 5, *D–E* and Fig. S5 and Table 1).

In combination with the T848 mutant, three FL mutations had no effect on voltage sensitivity of the channel (Fig. S5 and Table 1). The F1432L mutant, however, caused an even stronger hyperpolarization in the voltage dependence of activation than what we measured in the I848T mutation alone (Fig. 5, *D–E* and Table 1). This result strongly suggests an interaction between F1432 and the predicted phosphate group at T848. This is supported by a predicted distance of only 3.6 Å between the benzene ring of F1432 and the phosphate group (Fig. S4D). Contrary to what we had predicted, it seems that the phenylalanine–phosphate interaction limited the negative shift in voltage sensitivity of the I848T mutant to −9.77 ± 1.45 mV (Fig. 5, *D–E* and Table 1). With this interaction taken away, the I848T channel now became even more hypersensitive.

In summary, we can see that a negatively charged side group (glutamate or phosphothreonine) seems to cause the hyperpolarized shift in the voltage dependence of activation and this shift appears to be limited by interactions with the neighboring residue F1432.

## Discussion

Patients with IEM have severe pain attacks, which can often reduce the quality of life, especially because there is no satisfying treatment for the disease (7, 14). Even though many mutations in Navs are known to cause IEM, we only have a limited understanding of the mechanisms that lead to the changes in channel gating and the increased pain sensations.

In our study, we show that phosphorylation of the IEM-causing Nav1.7/I848T mutant by PKC leads to a hyperpolarized shift in the voltage dependence of activation and therefore to hypersensitivity of Nav1.7, causing a gain in pain.

### I848T induces a hyperpolarized shift in the voltage dependence of activation

As one of the early described mutations causing IEM, the I848T mutation has been the subject of several studies (6, 15, 16, 17). Recently, it was shown that the location of the I848T mutation within the S4–S5 linker of domain II may be structurally important for the opening and functioning of the channel (29, 40, 41). This notion is supported by the fact that the isoleucine at I848 is highly conserved throughout almost all vertebrate species (Fig. 6). Accordingly, mutations at this location could cause changes in the voltage dependence of activation and lead to, *e.g.*, hypersensitivity.

Similar to earlier studies, we have shown a hyperpolarized shift in the voltage dependence of activation for the I848T mutation (6, 16, 17). We also noticed a decrease of the current density for the I848T mutant, which is contrary to previous findings, both in heterologous expression systems (17) and in iPS-derived sensory neurons (6). However, it should be noted that the former study had cotransfected the β1- and β2-subunit together with Nav1.7, which can affect the gating and trafficking of the channel (1) and which would also mimic the situation in sensory neurons more closely. Therefore, we repeated our experiments with the cotransfected β1- and β2-subunits together with Nav1.7. In general, current density was increased for both, the wildtype and I848T mutant channels (Fig. S1*C* and Table S1), supporting an effect of β-subunits on Nav trafficking. In fact, an increased current density of Nav1.7 owing to especially β1 has been described before (42). However, these experiments fully reproduced the hyperpolarized shift and the decrease in current density for the I848T mutant.

We do not believe that the reduced current density necessarily represents a loss of function, which counteracts the gain of function of the increased voltage sensitivity. First, the current density in heterologous expression systems can be affected by the overexpression of the channel owing to transient transfection. Second, the current density heavily depends on the cell type and will be very different in neuronal cells. Indeed, we do not see a reduced current density in iPS-derived nociceptors of patients carrying the I848T mutation (6). Finally, it seems that only a very small fraction of Nav channels is required to induce action potential firing (6).

We did not find an impact of the mutation on steady-state fast inactivation, which is in line with the results from the aforementioned study (17), but is contrary to the results of a later study (16), which used Chinese hamster ovary cell line (CHO-K1) cells rather than HEK293 cells. Using different expression systems could explain the different outcomes (16, 17). Therefore, the neuronal hyperexcitability induced by the mutation (6, 15, 19) seems mainly to be caused by the hyperpolarized shift in the voltage dependence of activation.

### A negatively charged residue changes channel function

We used the negatively charged residue glutamate to mimic the negative charge of the putative phosphothreonine. This I848E mutation in fact showed a similar behavior in the voltage dependence of activation compared with the I848T mutant. The hyperpolarized shift was smaller than the one of I848T, but it supports the idea that a negative charge at this position could be the reason for the change in the voltage dependence of Nav1.7. A possible explanation for the smaller effect of I848E compared with I848T is that, at physiological pH, glutamate only has a single negative charge, whereas a phosphothreonine maintains a double negative charge (35, 43). The second negatively charged mutant I848D, did not produce any current. Since aspartate bears a smaller side chain than glutamate, we assume that this amino acid undergoes different molecular interactions with neighboring residues and therefore renders the channel nonfunctional.

### I848T is phosphorylated by PKC

We identified PKC to cause the phosphorylation of T848. In previous studies it had already been shown that phosphorylation can have an effect on Nav channels and a few phosphorylation sites were investigated (30, 32, 34). It was further speculated that such posttranslational modifications of Navs are involved in chronic pain syndromes and that phosphorylation of Nav1.7 might be important for the initiation of hypersensitivity (33). Especially the intracellular loop between domain I and II seems to be important for phosphorylation (31, 32, 34). In a similar way, the intracellular location of the T848 residue, situated in the S4–S5 linker of domain II, makes it accessible to kinases. This Nav1.7 mutant therefore creates a novel phosphorylation site in the channel protein.

Using staurosporine, a nonspecific kinase inhibitor, and calphostin C, a PKC selective inhibitor, we show that PKC is responsible for the phosphorylation at T848. In contrast, using PMA, a PKC activator, we were not able to further increase the hyperpolarized shift in the voltage dependence of activation for the I848T mutant. This suggests that the majority of channels are already phosphorylated at T848 under baseline PKC activity. Contrary to earlier findings in *Xenopus* oocytes, neither calphostin C nor PMA affected voltage sensitivity of wildtype Nav1.7 (30). This discrepancy is likely due to the different expression systems. Phosphorylation and kinase activity in general can be assumed to be very differently regulated in *Xenopus* oocytes as opposed to human HEK293 cells.

The current density in the wildtype channel was not affected by kinase modulation in our experiments. This is in contrast to one earlier study that showed a decrease in the current density in *Xenopus* oocytes for cells treated with a PKA activator (30). The same study also described a decreased peak current using PMA, which was prevented by the usage of calphostin C in *Xenopus* oocytes (30). We were not able to confirm these changes in the current density for Nav1.7 WT in a human expression system. Again, we assume that these conflicting results are due to the species difference in expression systems. In addition to kinase activity, ion channel trafficking and membrane expression are also differently regulated in these cell systems.

### Phosphorylated T848 interacts with surrounding phenylalanines

The position of the I848T mutation at the S4–S5 linker seems to be crucial for the gating of Nav1.7 (29, 40). The recent cryo-EM of the ancestral bacterial NavAb channel (29) identified three amino acid residues that are key for opening and closing of the channel gate: I119, L123, V126. The corresponding residue for L123 in human Nav1.7 domain II is I848, which may suggest that this residue is one of the crucial amino acids for channel gating. It is therefore likely that the I848T mutation interferes with the gating process especially considering that a change of the electric charge might change the possible interactions necessary for the gating process.

We identified four phenylalanines in close proximity to the phosphorylated T848 residue. The distance is close enough for most of the benzene rings to cause π-interactions (38, 39). Of interest, all four phenylalanines are highly conserved throughout all human Navs and throughout a few other species (Fig. 6). This suggests that these phenylalanines serve an important function and, furthermore, that any molecular interactions with these residues might easily affect channel function.

The nonfunctionality of the I848K and I848R mutants might emphasize the importance of the 848 locus for proper channel function and provides further support for a possible interaction of a predicted phosphate group at T848 with the surrounding phenylalanines: A negative charge at the 848 locus (either I848E or phosphorylated T848) might undergo weak interactions with the surrounding phenylalanines. This could be either in the form of an anion–π-interaction of the negative charge of the 848 locus with the partially positive carbon atoms of the edge of the phenylalanine aromatic ring or in the form of a repelling of the negative 848 residue by the partially negative face of the phenylalanine aromatic ring (44). This interaction reduces channel function but leaves a window for channel activity that would be subject to an altered voltage sensitivity. A positive charge at the 848 locus, however, would undergo stronger interactions with the surrounding phenylalanines, since cation–π-interactions are one of the strongest noncovalent interactions (39, 44). It is conceivable that such strong interaction would render the channel completely nonfunctional.

How can we explain the altered voltage sensitivity due to the inserted negative charge? In the case of the I848E and I848T mutants, it would be conceivable that a negative charge at location 848 would be repelled by the face of the π system of any of the surrounding phenylalanines (44), leading to greater flexibility of the S4–S5 linker and to a reduced constriction of the channel pore (29). This in turn might facilitate pore opening in a way that less energy (*i.e.*, smaller depolarization) is required to relieve the channel pore from the constriction. The result would be an increased voltage sensitivity, which others and we have shown for the I848T mutant (6, 16, 17). A positive charge at position 848, however, would lead to a positive interaction with the surrounding π systems, especially with F1436, reducing flexibility of the S4–S5 linker and strengthening the constriction of the channel pore, thus essentially stabilizing the closed state and abolishing channel function.

In conclusion, we have shown that phosphorylation of the mutated voltage-gated sodium channel Nav1.7/I848T can lead to a hyperpolarization in the voltage dependence of activation. This increases voltage sensitivity of the channel and may lead to the gain in pain sensations of the patients. This phosphorylation is caused by PKC but not PKA. We propose that the negative charge introduced by phosphorylation of T848 can undergo π-interactions with surrounding phenylalanines, which might mediate the change in voltage sensitivity. These findings suggest that targeting phosphorylation of Nav channels could provide a new avenue for the development of novel treatment options for chronic pain.

## Experimental procedures

### Cell culture

Cells from the human embryonic kidney cell line HEK293T were maintained in Dulbecco's modified Eagle's medium (Gibco–Life Technologies) including 10% fetal bovine serum, 1.0 gl^−1^ glucose, and 1% Geneticin (G-418) (Carl Roth). All cells were kept at 37 °C and 5% CO_2_. Cells were transfected with 1.25 μg Nav1.7-wildtype or Nav1.7-mutant DNA and 0.25 μg GFP using 3 μl JetPEI (Polyplus Transfections) according to the manufacturer's instructions. Cells were recorded 1 to 2 days after transfection.

For experiments with the β-subunits 0.9 μg Nav1.7-wildtype or Nav1.7-mutant DNA, 0.4 μg CD8 tagged β2, and 0.2 μg GFP-tagged β1 and 3 μl JETPEI (Polyplus Transfections) was used according to the manufacturer's instructions.

### Plasmids, DNA cloning, and mutagenesis

The mutations were generated in pCMV6neo-hNav1.7. For the mutations I848D and I848E, we used the site-directed Mutagenesis kit (NEB) using back-to-back primers (Eurofins), followed by kinase-ligase-*DpnI* treatment. For the other mutations, we did a sited-directed mutagenesis PCR using Q5 polymerase (NEB) and overlapping primers (Eurofins), followed by *DpnI* (Agilent) digestion. After kinase-ligase-*DpnI* treatment or *DpnI* digestion, plasmids were amplified by XL-gold bacteria (Agilent) followed by DNA extraction (ZymoPUREII, Plasmid MaxiprepKit, ZymoResearch). The β1- (pCLH-hSCN1B-EGFP) and β2- (pCLH-hSCN2B) plasmids were a kind gift by Prof Lerche (Department of Neurology and Epileptology, Hertie Institute for Clinical Brain Research, University of Tübingen) (45). All plasmids were checked for the respective mutation by sequencing (Eurofins).

### Electrophysiology

Whole-cell voltage-clamp experiments of transfected cells were performed using an EPC-10USB amplifier (HEKA Elektronik GmbH) at room temperature (21–23 °C). Glass pipettes (tip resistance between 0.9 and 1.7 MΩ) were manufactured with a DMZ puller (Zeitz Instruments GmbH) and filled with internal solution containing (in mM): NaCl 10, CsF 140, EGTA 1, Hepes 10, sucrose 18 (pH 7.33). The extracellular bath solution was prepared with (in mM): NaCl 140, KCl 3, MgCl_2_ 1, CaCl_2_ 1, Hepes 10, glucose 10 (pH 7.4).

Capacitive transients were cancelled and series resistance (always <6 MΩ) was compensated by at least 60%. Leak currents were subtracted online using the *P*/4 procedure following the test pulses. Signals were digitized at a sampling rate of 100 kHz. The low-pass filter frequency was set to 10 kHz. Voltage protocols were carried out after current stabilization over 3 min, during which the cells were held at −120 mV and stimulated by a 100-ms test pulse to 10 mV at 0.1 Hz.

For all voltage protocols, the holding potential was set to −120 mV. The voltage dependence of activation was assessed from holding potential using 40- or 100-ms pulses to a range of test potentials from −90 to +40 mV in 10-mV steps with an interval of 5 s. The current density was obtained from these protocols by dividing the maximum peak current by the cell capacitance. The voltage-dependent Nav conductance *G*_Na_ was calculated using the following equation: *G*_Na_ =*I*_Na_/(*V*_m_ − *E*_rev_), where *I*_Na_ is the peak of the current at the voltage *V*_m_ and *E*_rev_ is the reversal potential for sodium, which was determined for each cell individually. Activation curves were derived by plotting normalized *G*_Na_ as a function of test potential and fitted with the Boltzmann equation: *G*_Na_ = *G*_Na,max_/(1 + exp [(*V*_m_ − *V*_1/2_)/*k*]), where *G*_Na,max_ is the maximum sodium conductance, *V*_1/2_ is the membrane potential at half-maximal activation, *V*_m_ is the membrane voltage, and *k* is the slope factor. The voltage dependence of steady-state fast inactivation was measured using a series of 500-ms prepulses from −140 to 0 mV in 10-mV steps, immediately followed by a 40-ms depolarization to 0 mV that served as a test pulse to assess the available noninactivated channels. The normalized peak inward current amplitude (*I*_Na_/*I*_Na,max_) at each test pulse is displayed as a function of the prepulse potential and fitted using the above Boltzmann equation.

### Chemicals

All chemicals were dissolved in dimethyl sulfoxide and stored in stock solutions at −20 °C or during experiments at 4 °C. Kinase inhibitors or activators were added into the bath solution before patching. The incubation time was at least 20 min for staurosporine and 30 min for calphostin C, H-89, and PMA.

Staurosporine was purchased at Alomone labs (Cat.#: S-350) and stored protected from light.

Calphostin C and H-89 were ordered at Cayman Chemicals (Item#: 15383 and 10010556, respectively). PMA (phorbol 12-myristate 13-acetate) was purchased at Stemcell Technologies (Cat.#: 74042).

### Sequence alignment and molecular modeling

All sequence alignments were performed using Jalview (46) or Lalign (47). For the alignment of the different human Nav isoforms, the following sequences were used (all National Center for Biotechnology Information [NCBI]): NP_001189364.1 (Nav1.1), NP_001035232.1 (Nav1.2), NP_008853.3 (Nav1.3), NP_000325.4 (Nav1.4), NP_932173.1 (Nav1.5), NP_055006.1 (Nav1.6), NP_002968.1 (Nav1.7), NP_006505.3 (Nav1.8), NP_001336182.1 (Nav1.9). For the species alignment of Nav1.7, the following sequences were used: NCBI NP_002968.1 (human), NCBI XP_028686638.1 (*Rhesus macaque*), NCBI NP_001104257.3 (cattle), NCBI XP_022270547.1 (dog), NCBI XP_019693767.1 (cat), NCBI NP_579823.1 (rat), GenBank AAI72147.1 (mouse), NCBI NP_571703.1 (zebrafish), UniProt D0E0C2 (cockroach), Protein Data Bank 6P6W (NavAb).

Molecular modeling was performed in Pymol (Version 2.0, Schrödinger, LLC) using the recently published cryo-EM structure of human Nav1.7 in complex with β-subunits 1 and 2, ProTx-II, and tetrodotoxin (Protein Data Bank: 6j8j; [37]). The structures of both β-subunits and the toxins were removed for simplicity.

### Data analysis

Data were analyzed and graphed using Fitmaster software (HEKA Elektronik GmbH), Igor Pro 6 (Wavemetrics), Prism 7 (GraphPad Software), and Corel Draw X6 (Corel Corporation). Unless otherwise stated, data are presented as mean ± SD with the difference of the means ± standard error of difference and the confidence interval of the difference given in the figure legends. For analysis of the I848E mutant (and I848E and β1- and β2-cotransfection), a digital offline filter of 3 kHz was used in order to reduce noise, as the current amplitude was strongly reduced. All other analyses were performed with no digital offline filter.

For statistical testing, groups larger than two were compared by a one-way ANOVA followed by Bonferroni post hoc analysis. Groups of two were compared by unpaired *t* test or Mann–Whitney test depending on the normal distribution. Significance in every case at least *p* <0.05, represented by ∗.

We conducted a post hoc power analysis of all statistically tested data using G∗Power 3.1.9.6 (HHU Düsseldorf). Power (1 − β err prob.) for all groups was >0.99 unless otherwise stated.

## Data availability

The datasets generated and analyzed during the current study are available from the corresponding author on reasonable request.

## Conflict of interest

The authors declare that they have no conflicts of interest with the contents of this article.
