# Supplementary material for: Phosphorylation of a chronic pain mutation in the voltage-gated sodium channel Nav1.7 increases voltage sensitivity
Source: J Biol Chem. 2020 Dec 29;296:100227. doi: 10.1074/jbc.RA120.014288 (PMC7948457; doi:10.1074/jbc.RA120.014288)
Supplement: Figures S1 to S7 and Table S1 [file mmc1.pdf]

Phosphorylation of a chronic pain mutation in the voltage-gated sodium channel Nav1.7 increases voltage sensitivity

Clara M. Kerth[1], Petra Hautvast[1], Jannis Körner[1,2], Angelika Lampert[1], Jannis E. Meents[1,#,]*

[1]Institute of Physiology, Uniklinik RWTH Aachen University, Pauwelsstrasse 30, 52074 Aachen, Germany;
[2]Department of Anesthesiology, Uniklinik RWTH Aachen University, Medical Faculty, Pauwelsstrasse 30, 52074 Aachen, Germany;

Supporting material:

Figure S1: Comparison of WT, IT and IE with and without the co-transfection of β-subunits.
Figure S2: Effects of kinase inhibitors on steady-state fast inactivation.
Figure S3: Current density for PKC inhibitor calphostin C and PKC activator PMA.
Figure S4: Modeling of the S4/S5-linker with different mutations.
Figure S5: Three phenylalanine residues do not seem to interact with the phosphorylated I848T.
Figure S6: Current-voltage relationship for the F1432L mutant
Figure S7: Calphostin C also reduces the hyperpolarized shift of the voltage dependence of activation of I848T in the presence of β subunits.

Table S1: Data of co-transfected channels with β1 and β2 subunit.

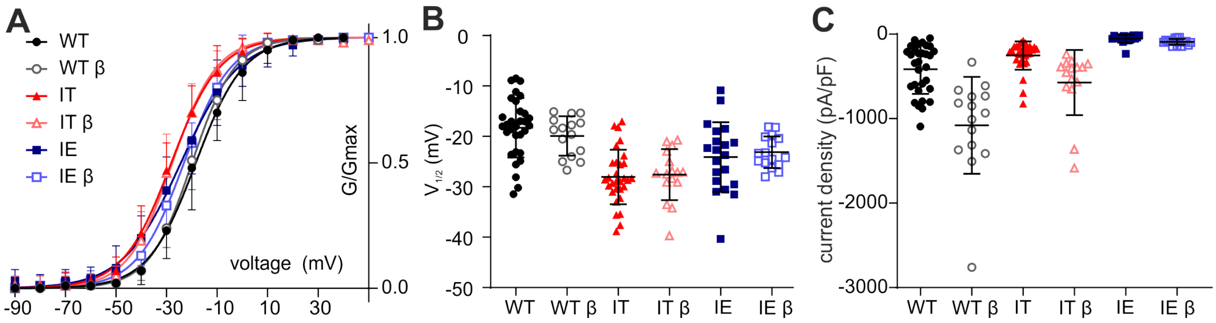

Figure S1. Comparison of WT, IT and IE with and without the co-transfection of β-subunits. (A) The co-transfection with β1 and β2 does not affect the voltage dependence of activation. (B) Mid-point of activation for WT, IT and IE with and without co-transfected β-subunits. (ANOVA F = 12.98; WTβ vs. ITβ p = 0.0031, mean diff. 7.57 +/- 1.98 mV, 95% CI 1.65 to 13.49 mV). Colour-coding is the same as in (A). (C) The co-transfection with β1 and β2 does affect the current density of WT and IT. However, the phenotype of a reduced current density for I848T and I848E still persists (ANOVA F = 28.49; WT vs. WTβ p <0.0001, mean diff. 663 +/- 90.86 pA/pF, 95% CI 390.9 +/- 935.1 pA/pF; IT vs. ITβ p = 0.0095, mean diff. 320 +/- 91.35 pA/pF, 95% CI 47.08 +/-594.2 pA/pF).
Data for WT, I848T and I848E are the same as in Fig. 1 and 3 and only presented for comparison. All 1-way ANOVA with Bonferroni multiple comparisons test. See Table 1 and S1 for all values. IT = I848T, IE = I848E, β = co-transfected with β1 and β2 subunit Post-hoc power analysis for data tested with G*Power provided the following values: 0.99 (B). All data presented as mean ± SD.

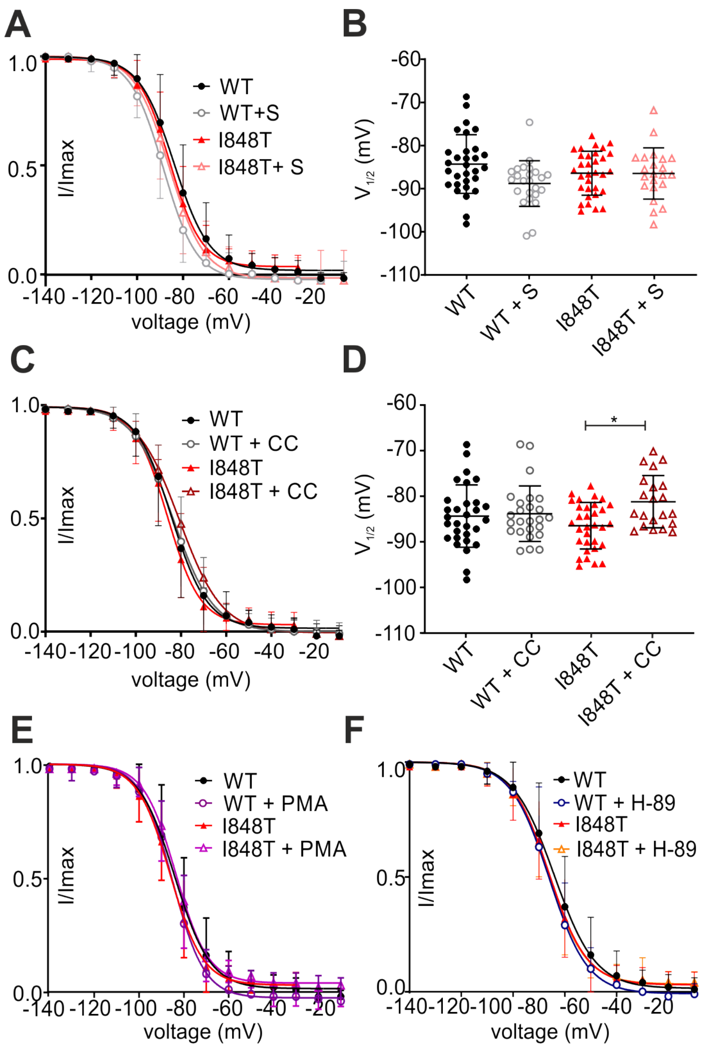

Figure S2. Effects of kinase inhibitors on steady-state fast inactivation.
(A) Steady-state fast inactivation of WT and I848T treated with 500 nM staurosporine, a non-specific kinase inhibitor. (B) $V_{1/2}$ of fast inactivation does not show a significant difference. (C) Steady-state fast inactivation of WT and I848T treated with 200nM of the specific protein kinase C (PKC) inhibitor calphostin C. (D) $V_{1/2}$ of fast inactivation for channels treated with calphostin C (ANOVA F = 3.356, I848T vs. I848T+C p = 0.0129, mean diff. -5.27 +/- 1.68 mV, 95% CI -9.78 to -0.767 mV). (E) Fast inactivation curves for channels treated with 1µM PMA (Phorbol 12-myristate 13-acetate), a specific activator of PKC. (F) Steady-state fast inactivation shown for WT and I848T treated with the specific PKA (protein kinase A) inhibitor H-89 (10µM). Data for WT, I848T and I848E are the same as in Fig. 1 and only presented for comparison. All 1-way ANOVA with Bonferroni multiple comparisons test. See Table 1 and Table 2 for all values. All data presented as mean ± SD. CC = calphostin C, S = staurosporine.

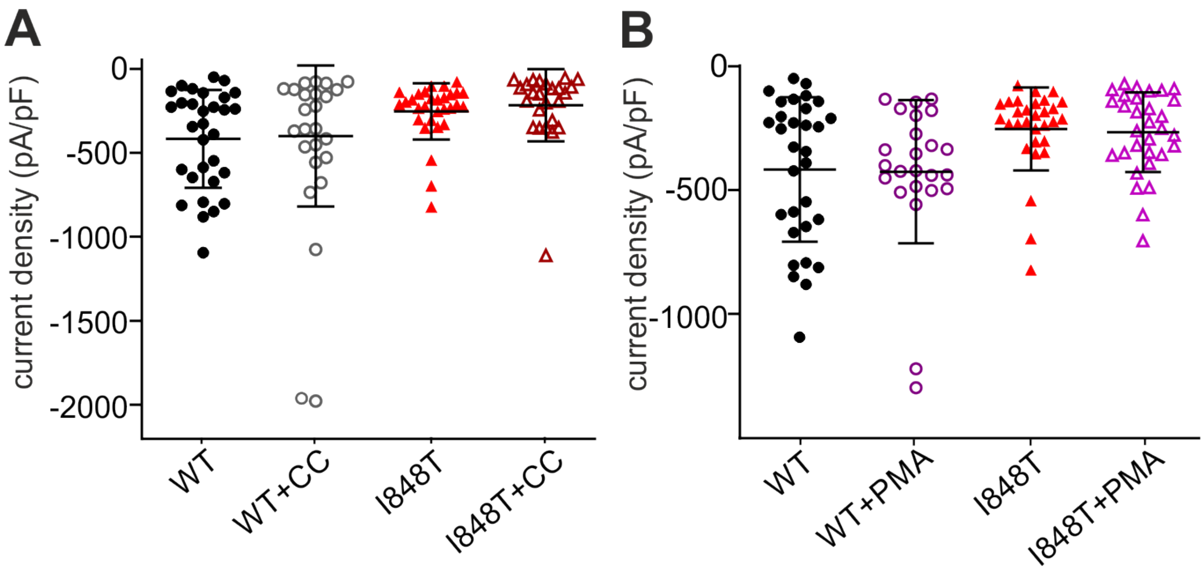

Figure S3. Current density for PKC inhibitor calphostin C and PKC activator PMA.
(A) Current density shown for I848T and WT with and without calphostin C (200nM). (B) PMA(1µM) does not change current density for WT and I848T. Data for WT and I848T are the same as in Fig. 1 and only presented for comparison. All 1-way ANOVA with Bonferroni multiple comparisons test. See Table 1 and Table 2 for all values. All data presented as mean ± SD. CC = calphostin C.

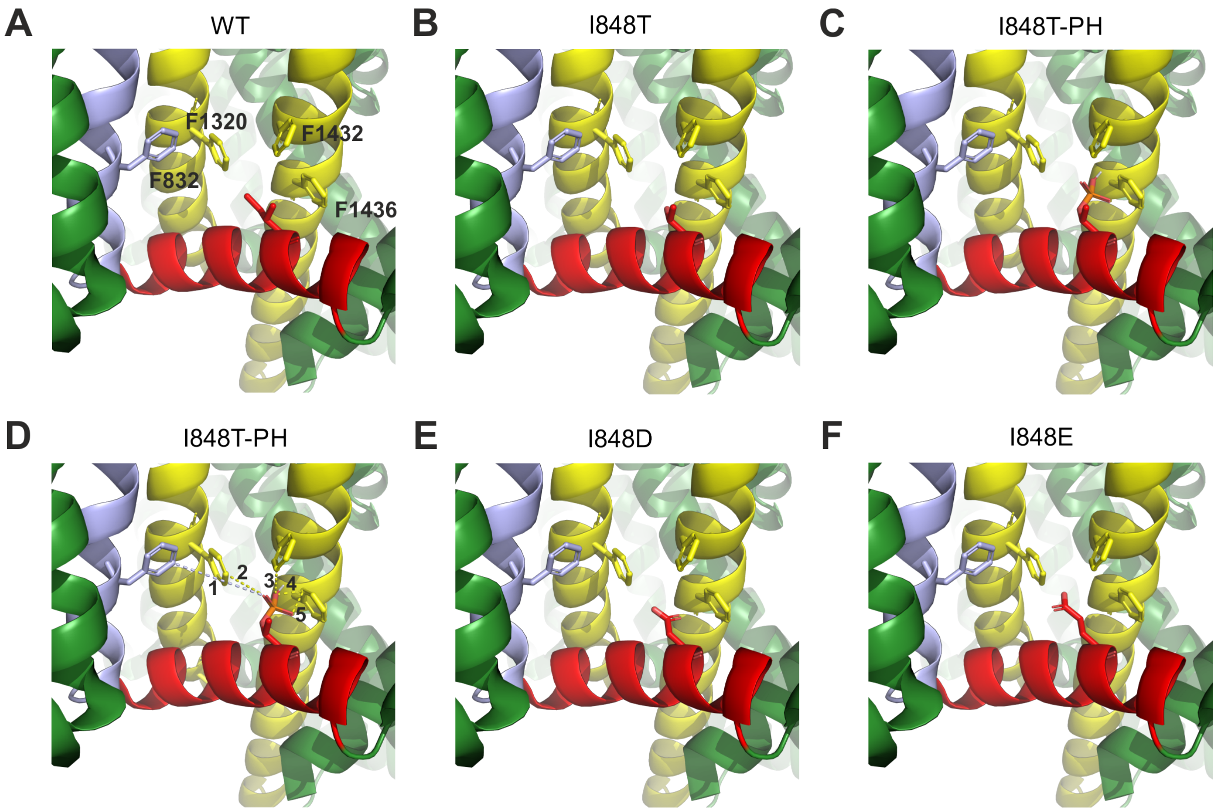

Figure S4. Modelling of the S4/S5-linker with different mutations.
(A-F) Magnified view of the S4/S5 linker of DII in human Nav1.7. The I848 residue or its respective mutation as well as the four surrounding phenylalanines are displayed as sticks. Colour coding of the

different segments is the same as in Fig. 5. (A) Wild type Nav1.7. (B) I848T mutation without phosphorylation. (C) Phosphorylated I848T mutant. (D) Phosphorylated I848T mutant with distances of the phosphate group to the four phenylalanines: 1 = 7.5 Å, 2 = 4.5 Å, 3 = 3.6 Å, 4 = 2.2 Å, 5 = 2.5 Å. (E) I848D mutant. (F) I848E mutant.

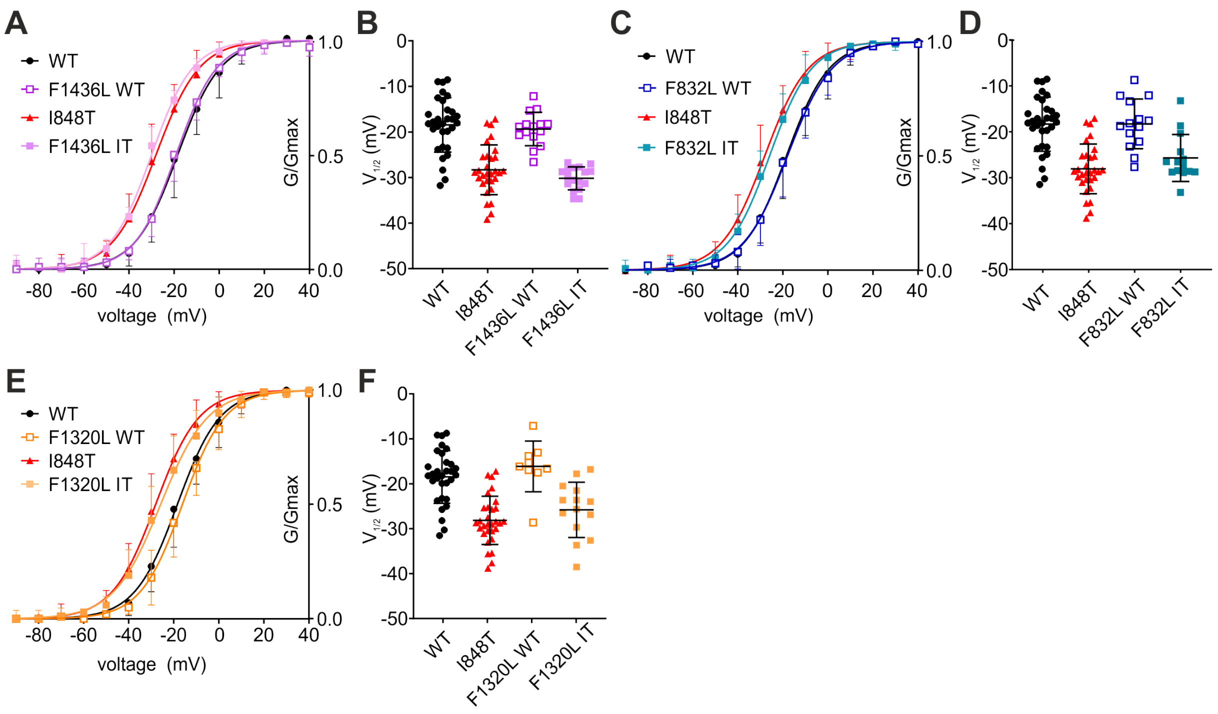

Figure S5. Three phenylalanine residues do not seem to interact with the phosphorylated I848T (A-B) Voltage dependence (A) and $V_{1/2}$ (B) of activation for F1436L expressed in a WT background (F1436L WT) and in combination with I848T (F1436L IT). (C-D) Voltage dependence (C) and $V_{1/2}$ (D) of activation for F832L expressed in a WT background or in combination with the I848T mutation. (E-F) Voltage dependence (E) and $V_{1/2}$ (F) of activation for F1320L expressed in a WT background or in combination with the I848T mutation. Data for WT, I848T and I848E are the same as in Fig. 1 and only presented for comparison. All 1-way ANOVA with Bonferroni multiple comparisons test. See Table 1 for all values. Post-hoc power analysis for data tested with G*Power provided the following values: 0.99 (B), 0.95 (D), and 0.97 (F). All data presented as mean ± SD.

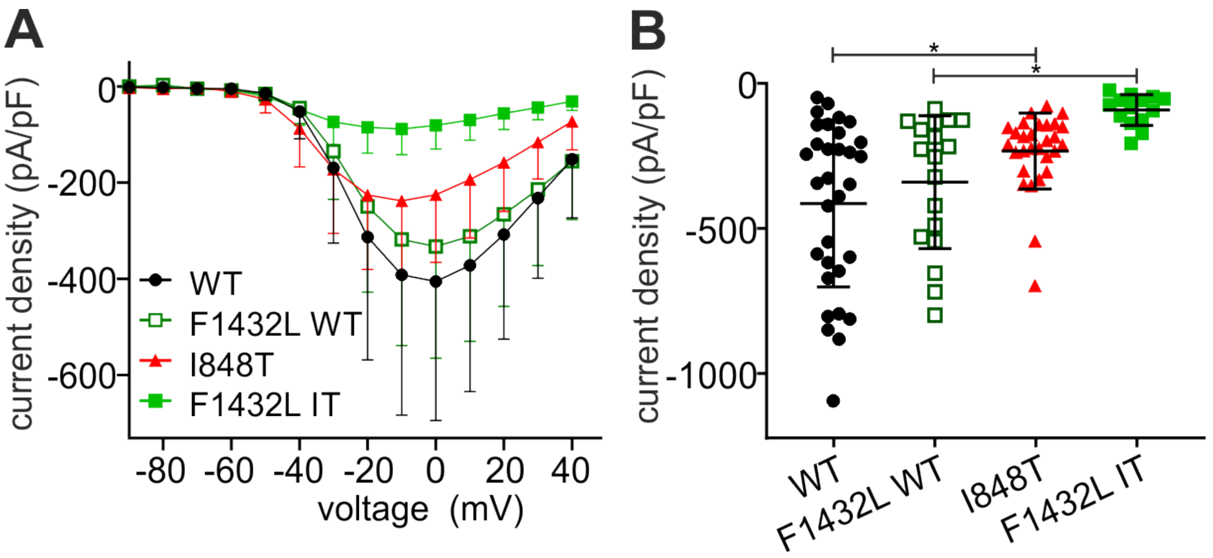

Figure S6. Current-voltage relationship for the F1432L mutant.

(A) F1432L IT produces less current than the I848T mutant for all voltages. Data for WT, I848T and I848E are the same as in Fig. 1 and only presented for comparison. (B) current density presented, showing a decreased current density for the I848T and the F1432L IT mutant. (ANOVA F =8.86, WT vs. I848T p = 0.0071, mean diff. -181.2 pA/pF, 95% CI -327.2 to -35.22 pA/pF; F1432L WT vs. F1432L IT p= 0.0081, mean diff. -248.7 pA/pF, 95% CI -451.6 to -45.82 pA/pF,) All 1-way ANOVA with Bonferroni multiple comparisons test. See Table 1 for all values.

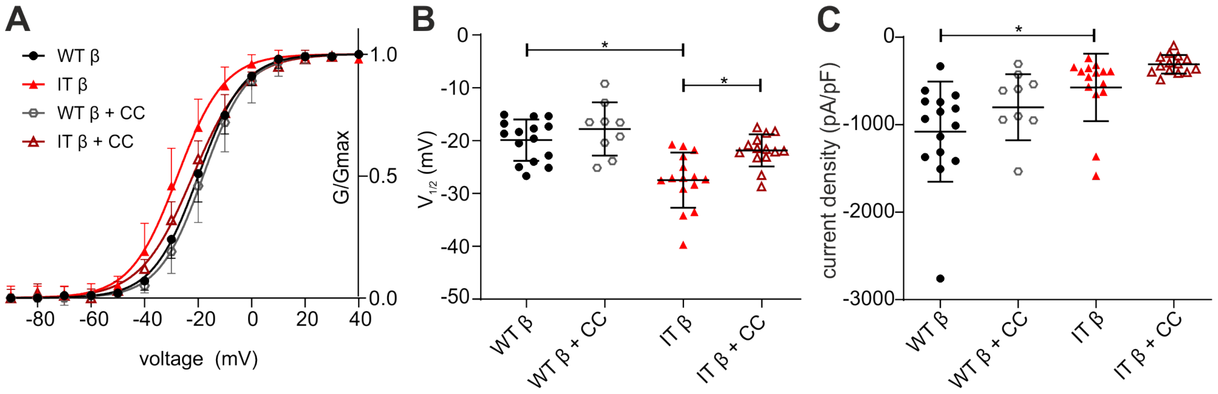

Figure S7. Calphostin C also reduces the hyperpolarized shift of the voltage dependence of activation of I848T in the presence of β subunits.

(A) The selective PKC inhibitor calphostin C (200 nM) also abolishes the hyperpolarized shift in the voltage dependence of activation for the I848T mutant when co-transfected with β1 and β2 subunits. (B) The voltage dependence of activation ($V_{1/2}$) is shown for co-transfected WT and I848T. (ANOVA F = 11.96; WTβ vs. ITβ p <0.0001, mean diff. -7.57 +/- 1.58 mV, 95% CI -11.92 to -3.23 mV; ITβ vs. ITβ + CC p = 0.0061, mean diff. -5.622 +/- 1.61 mV, 95% CI -10.05 to -1.2 mV; IT β vs. WT β + CC p <0.0001, mean diff. -9.67 +/- 1.83 mV, 95% CI -14.69 to -4.66 mV). Colour-coding is the same as in (A). (C) Current density shown for calphostin C treated co-transfected WT and I848T mutant. (ANOVA F = 9.53; WTβ vs. ITβ p = 0.0071, mean diff. -506 +/- 146.9 pA/pF, 95% CI 909.8 to -102.2 pA/pF; WTβ vs. IT β + CC p <0.0001, mean diff. -770.6 +/- 149.5 pA/pF, 95% CI -1182 to -359.7 pA/pF) Colour-coding same as in (A). All 1-way ANOVA with Bonferroni multiple comparisons test.

See Table S1 for all values. All data presented as mean ± SD. β = co-transfected with β1 and β2 subunit. PKC = protein kinase C, CC = calphostin C, IT = I848T. Post-hoc power analysis for data tested with G*Power provided the following values: 0.99 (B).

	Activation		Current density
	**n**	**$V_{1/2}$ (mV)**	**(pA/pF)**
**WTβ**	15	-19.92 ± 3.91	-1079 ± 572.8
**I848Tβ**	15	-27.49 ± 5.22	-573.4 ± 385.4
**I848Eβ**	16	-23.15 ± 3.13	-91.94 ± 34.66
**WTβ + CC**	9	-17.82 ± 5.04	-800.1 ± 119.5
**ITβ + CC**	14	-21.87 ± 3.04	-308.8 ± 28.3

Table S1. Data of co-transfected channels with β1 and β2 subunit.
All data is presented as mean ± SD. Calphostin C (CC) is a PKC inhibitor. 200nM calphostin were used for the experiments. β = co-transfected channels with β1 and β2 subunit. CC = calphostin C.
